# Mapping genes governing flower architecture and pollen development in a double mutant population of carrot

**DOI:** 10.3389/fpls.2014.00504

**Published:** 2014-10-08

**Authors:** Holger Budahn, Rafał Barański, Dariusz Grzebelus, Agnieszka Kiełkowska, Petra Straka, Kai Metge, Bettina Linke, Thomas Nothnagel

**Affiliations:** ^1^Institute for Breeding Research on Horticultural Crops, Federal Research Centre for Cultivated Plants, Julius Kühn-InstituteQuedlinburg, Germany; ^2^Department of Genetics, Plant Breeding and Seed Science, Faculty of Horticulture, University of AgricultureKraków, Poland; ^3^Institute for Biosafety in Plant Biotechnology, Federal Research Centre for Cultivated Plants, Julius Kühn-InstituteQuedlinburg, Germany; ^4^Department of Biology, Humboldt UniversityBerlin, Germany

**Keywords:** *Daucus carota*, linkage map, MADS-box genes, male gametogenesis, alternative oxidase, chalcone synthase

## Abstract

A linkage map of carrot (*Daucus carota* L.) was developed in order to study reproductive traits. The F_2_ mapping population derived from an initial cross between a *yellow leaf* (*yel*) chlorophyll mutant and a *compressed lamina* (*cola*) mutant with unique flower defects of the sporophytic parts of male and female organs. The genetic map has a total length of 781 cM and included 285 loci. The length of the nine linkage groups (LGs) ranged between 65 and 145 cM. All LGs have been anchored to the reference map. The objective of this study was the generation of a well-saturated linkage map of *D. carota*. Mapping of the *cola-locus* associated with flower development and fertility was successfully demonstrated. Two MADS-box genes (*DcMADS3*, *DcMADS5*) with prominent roles in flowering and reproduction as well as three additional genes (*DcAOX2a*, *DcAOX2b*, *DcCHS2*) with further importance for male reproduction were assigned to different loci that did not co-segregate with the *cola*-locus.

## Introduction

Carrot is the second most economically valuable vegetable in the European Union and is also of worldwide significance. Increased production area and improved productivity highlight its economical importance in North America, Asia, and Europe (*http://faostat.fao.org/*). Over the last few decades, carrot consumption has continuously grown (Simon et al., 2009).

*Daucus carota* is a typical biannual diploid (2n = 2x = 18) outcrossing species with a relatively small genome estimated as 473 Mb. Carrot chromosomes are small but morphologically distinguishable (Schrader et al., 2003). Within the large family of Apiacea, carrot is the best characterized species at the genetic and molecular level. Comprehensive research has been performed on the characterization of metabolite contents, morphological traits and resistances (for review see Brandeen and Simon, 2007; Simon et al., 2008). On the other hand, knowledge on genes controlling reproduction and flowering is yet limited. To facilitate genetic characterization by improved mapping studies, several tools have been made available in recent years. This includes a deep coverage BAC library (Cavagnaro et al., 2009), assignment of linkage groups (LGs) to chromosomes by fluorescence *in situ* hybridization (Iovene et al., 2011) and *de novo* assembly of the carrot transcriptome (Iorizzo et al., 2011) and opens advanced perspectives for carrot research and breeding.

Improved knowledge on genetic control mechanisms for plant reproduction traits such as flowering time, fertility and seed set is of essential importance for the breeding process, especially for the stabilization of seed yield under different environmental conditions. One of the key steps is the determination of the basal floral architecture, i.e., the specification of sepals, petals, stamens, and carpels. Based on studies in model plants like *Arabidopsis* and *Antirrhinum*, the ABC model of flower development was proposed (Coen and Meyerowitz, 1991). The model was further extended by D- and E-class genes to include the advanced development of reproductive organs (Pelaz et al., 2000; Theissen, 2001). The majority of these genes belong to the MADS-box gene family of transcription factors (Schwarz-Sommer et al., 1990). MADS-box genes can be grouped into different clades with subfamily-specific functions in flower development (Becker and Theissen, 2003; Parenicova et al., 2003). Genes of these groups play a critical role in the determination of the identity of two neighboring flower whorls according to the ABCDE model. In the carrot, Linke et al. (2003) have identified five MADS-box genes (*DcMADS1* to *5*) and assigned them to the previously defined groups SQUAMOSA, GLOBOSA, DEFICIENS, AGAMOUS, and SEPALLATA1.

Other classes of genes are involved in the development of functional pollen and are important for fertility/sterility. The alternative oxidase (AOX) is a mitochondrial ubiquinol:oxygen oxidoreductase (Affourtit et al., 2002). It is encoded by a small nuclear multigene family (*AOX1* and *AOX2*). For the gene *AOX2b* a striking effect on male and female gametogenesis was shown in soybean. Its regulation depends on the tissue and the developmental stage (Finnegan et al., 1997; Djajanegara et al., 2002). A significant effect on pollen abortion and pollen germination was measured (Chai et al., 2010). In the pistils of transgenic antisense *GmAOX2b* soybean plants, a higher portion of immature-sized and non-fertile embryo sacs were observed. In addition, significant changes of the expression level for alternative oxidase in maize mitochondrial mutants were identified (Karpova et al., 2002).

Another enzyme with effects at particular stages of anther and pollen development is chalcone synthase (CHS), one of the key enzymes involved in the flavonoid biosynthesis pathway. Flavonoids are important for pollen development and plant fertility in several plant species (Dobritsa et al., 2010 and references therein). *CHS* has been reported as a potential sex-determination gene that is expressed in male cones of *Pinus* (Walden et al., 1999) and in male flower buds of *Silene* (Ageez et al., 2005). Significant effects of CHS on different types of nuclear male sterility were demonstrated for maize (Mo et al., 1992), petunia (Taylor and Jorgensen, 1992; van der Meer et al., 1992) and tobacco (Atanassov et al., 1998). In plants carrying the cytoplasmic male sterility trait (CMS), the deficiency of flavonoids caused by inhibition of *CHS* expression was strongly associated with pollen abortion (Yang et al., 2008). A role of *CHS* genes in the formation of the exine during microspore development has been reported in *Arabidopsis* (Dobritsa et al., 2010).

The first well-saturated map of all nine carrot LGs was generated by Just et al. (2007). Transposon display (td) markers were integrated into that map by Grzebelus et al. (2007) using *DcMaster*, a family of *PIF/Harbinger*-like transposable elements identified in *Daucus*. Simple sequence repeats (SSRs) have great advantages in comparison to other marker classes and were applied in research on carrot gene flow and genetic diversity (Rong et al., 2010; Barański et al., 2012). High reproducibility, abundance and a codominant inheritance make SSR markers particularly well suited for anchoring different maps of the same or closely related species. Niemann et al. (1997) have developed the first set of SSR markers for carrot and later assigned 19 SSR markers to a carrot linkage map (Niemann, 2001). Other sets of SSR markers were described by Umehara et al. (2005) and Clotault et al. (2010). By far more SSR markers became available to anchor new *D. carota* linkage maps, when Cavagnaro et al. (2011) provided a set of 300 SSR markers for carrot and assigned 55 of them to all nine LGs. In parallel, Iorizzo et al. (2011) has described a set of SSR markers from transcribed sequences (eSSR). These sets of SSR markers were used by Alessandro et al. (2013) for mapping *Vrn1*, a gene controlling early flowering and *Rf1*, a restorer gene for the petaloid-type of CMS in carrot. Yildiz et al. (2013) reported the mapping of anthocyanin biosynthesis genes on five individual carrot LGs. Recently, a carrot map on the base of Diversity Array Technology (DArT) markers became available (Grzebelus et al., 2014).

Due to the high genetic diversity within the carrot with substantial allelic variability (Macko and Grzebelus, 2008; Grzebelus et al., 2014), an extended genetic map will be advantageous to allocate and improve genetic information and to facilitate the possibility to set up meta-analyses of favorable genetic traits (Brachi et al., 2011). The objective of the present study was the generation of a well-saturated linkage map of *D. carota* comprising markers linked to known and new loci associated with flower development and fertility.

## Materials and methods

### Plant material

**Figure 1:**
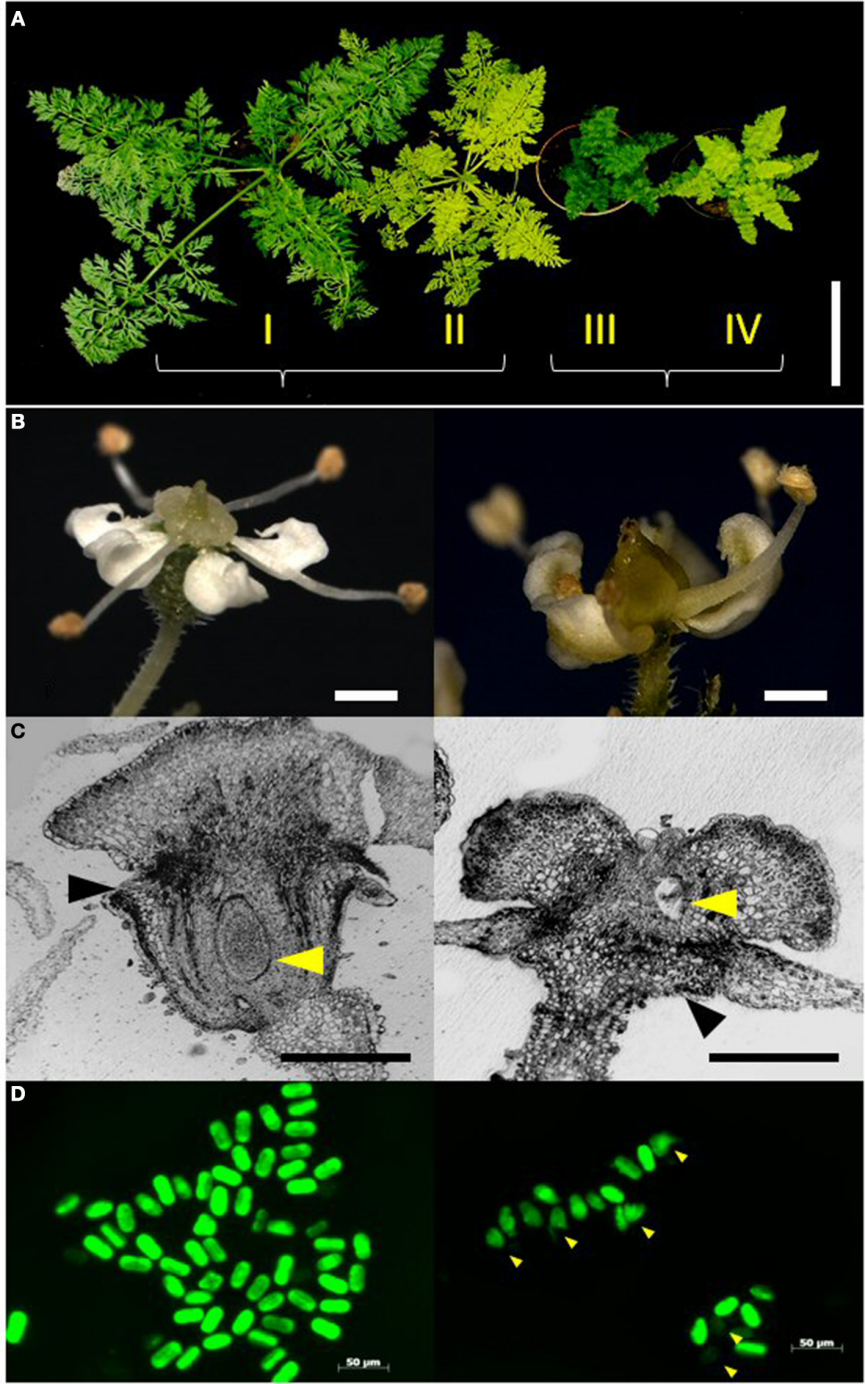
**Characterization of the phenotype classes. (A)** Representative plants of the four phenotype classes I–IV in the F_2_ segregating population DM19 obtained from a cross between mutants *yel* and *cola*. From left to right: wild-type plant with green and normal leaf structure (I); *yel*-mutant (II); *cola*-mutant (III); *yel/cola* double mutant (IV) (Bar = 10 cm). **(B)** Morphology of reproductive organs. Left: epigynous flower of wild-type. Right: hypogynous flower of *cola*-mutant (Bars = 1.0 mm). **(C)** Histo-morphological sections of flowers. Left: wild-type. Right: *cola*-mutant. Black arrowheads: petals, yellow arrowheads: ovule (Bars = 0.5 mm). **(D)** FDA pollen staining. Fertile pollen grains revealed a intensive greenish stain, whereas sterile and undeveloped pollen grains were stained weakly; Left: wild-type, Right: *cola*-mutant, arrowheads indicate undeveloped microspores.

The F_2_ mapping population DM19 was developed from an initial cross of the homozygous recessive *yellow leaf* (*yel yel*) mutant as the maternal parent and the *compressed lamina* (*cola cola*) mutant as the pollen parent. The *yel*-mutant is easily distinguishable from the wild-type by the yellow leaf color due to chlorophyll deficiency (Nothnagel and Straka, 2003). The *cola*-mutant is characterized by compact small leaves. The flower architecture of *cola*-plants did not correspond to one of the major “homeotic” phenotypes as described by the ABCDE model indicating that the reproductive organs have acquired a correct identity. Instead, rather advanced stages of reproductive organ differentiation were impaired. The female organs revealed a slight, but unique difference of the ovary position that has changed from an inferior position (epigynous flower type) to a rather superior position (hypogynous flower type) as was shown in Figure 1C. Thus, the ovary of *cola*-mutant flowers was located above the insertion point of the perianth-ring. This trait is always associated with the trait “anther/locule defect” of the *cola*-type flowers, indicating an irregular structure of several theca and/or locules as was previously shown by histo-morphological investigation (Nothnagel et al., 2005). For flower characterization of the mapping population we have used the trait “epigynous/hypogynous” because it is easy to characterize and do not require additional histological analyses. Hence, the *cola*-mutant flowers revealed defects of the sporophytic parts of both, male and female organs as a result of an impaired advanced differentiation. In contrast, the *yel*-parent of the mapping population revealed a wild-type phenotype for flower architecture.

Plants were grown in 16 cm plastic pots in a sand-humus mixture (v/v 3/1) in the greenhouse. To induce flowering, the plants were vernalized in a climatic chamber at 5°C for 12 weeks in the dark. Flowering plants were isolated and crossed manually to produce F_1_ plants (*YEL yel COLA cola*). True hybrids expressed the wild-type phenotype and were self-pollinated to produce F_2_ seeds. Leaf color and leaf shape of the F_2_ plants were evaluated 30, 60, and 90 days after sowing. Bolting status of the F_2_ plants was observed visually approximately each 5 days after vernalization and replantation in the glasshouse. The flower architecture was investigated using a stereoscopic microscope.

For the statistical analyses was used the software Systat 13 (Chicago, IL: Systsat Software, Inc., 2009). Data of F_2_- segregation were analyzed using the χ^2^-test to determine the goodness of fit for Mendelian inheritance (9:3:3:1 or 3:1, respectively).

### Analysis of pollen viability

The pollen viability was determined by vital staining of microspore cells, using fluorescein diacetate (FDA) according to Heslop-Harrison and Heslop-Harrison (1970). From ten plants of the *cola*-mutant and ten plants of the wild-type, five protandrous flowers were collected and the anthers were immediately suspended in 0.5 ml of a solution containing 75 mg/ml sucrose and 1 mg/ml FDA (Serva, Heidelberg, Germany). Pollen viability was determined as ratio of pollen emitting strong fluorescence when subjected to UV light using Axioskop 2 (Zeiss, Oberkochen, Germany) equipped with a fluorescence filter set 44 [excitation BP 475/40 (455–495 nm)/emission BP 530/50 (505–555 nm)]. At least 400 pollen grains per plant were classified. The mean values of pollen analysis data were compared by Student's *t*-test at a significance level *p* < 0.05.

### DNA extraction, RAPD-, double primer (dp)RAPD- and AFLP-analysis

Genomic DNA was extracted from 0.5 g of young leaf tissue from both parental lines and from 161 F_2_ plants of the DM19 population, using the method of Porebski et al. (1997). RAPD-PCR reactions were performed in 10 μl volume containing 1 x PCR buffer (InVitek, Berlin, Germany), 2.25 mM MgCl_2_, 0.2 mM dNTPs, 0.2 μM arbitrary decamer primer (Roth GmbH, Karlsruhe, Germany), 0.12 U InViTaq-polymerase (InVitek) and 8 ng total plant DNA. The thermocycler (PT 200; Bio-Rad, Hercules, CA, USA) was programmed as follows: initial denaturation at 94°C for 2 min; 45 cycles of 94°C for 1 min, 36°C for 1 min, 72°C for 2 min and final extension at 72°C for 7 min. Amplification products were separated on 1% agarose gels using the 100 bp ladder (Invitrogen, Carlsbad, CA, USA) for size determination. The generation of dpRAPD markers followed the protocol described by Budahn et al. (2008). AFLP reactions were performed according to the instructions of the AFLP® analysis system I kit (Invitrogen, Carlsbad, CA, USA). The amplification products were separated at 50°C and 100 W for 3.5 h on 4% denaturating polyacrylamide gels (Sequigen GT; 38 × 50 cm; Bio-Rad Laboratories, Hercules, CA, USA) followed by silver staining according to Bassam et al. (1991). Fragment sizes were estimated using the 123 bp DNA-ladder and the 1D-phoretix-Software 5.2 (Biostep, G, Nonlinear Dynamics Ltd., Newcastle, UK). Only unambiguous and polymorphic fragments were scored. The designation of the RAPD markers contained the used decamer primer sequence and, the AFLP markers the applied *Eco*RI- and *Mse*I-primers added by the estimated fragment sizes.

### SSR markers

The original primers and amplification conditions were described by Cavagnaro et al. (2011). A universal fluorescent labeling strategy was used, as described by Schuelke (2000). The unlabeled forward primer extended at the 5′ end by the 19 bp M13 sequence was combined with the original reverse primer and the fluorescently labeled M13 universal primer. The amplification products of two independent reactions with IRDye700 and IRDye800 labeled M13 primers were mixed. DNA fragments were separated on 6.5% polyacrylamide gels followed by fragment detection using a LI-COR 4300 automatic sequencer (LI-COR Biosciences, Lincoln, NE, USA). The fragment sizes were calculated by IRDye700 and IRDye800 labeled 50–350 or 50–700 bp size ladders, respectively. SSR markers of the DCM series (Niemann, 2001) were amplified, separated on the Sequigen GT system (Bio-Rad Laboratories, Hercules, CA, USA) and visualized by silver-staining. Primer sequences and annealing temperatures were summarized in Table S1.

### td markers

The primer sequences and amplification conditions have been described by Grzebelus et al. (2007). Ten primer combinations were selected for the analysis. The amplification products were separated on 4.5% polyacrylamide gels and visualized by silver staining (Bassam et al., 1991).

### Gene-specific markers

MADS-box genes of the carrot have been identified after generation of a cDNA library from wild-type carrot flowers (Linke et al., 2003). Additional sequences of genes with expected roles during reproductive organ differentiation and pollen development in the carrot were selected from NCBI database (complete list in Table S1; short list in Table 1). Gene-specific primers for PCR and mapping analyses were generated in the frame of earlier work (Linke et al., 2003; Campos et al., 2009; Cardoso et al., 2009; Grzebelus, pers. commun.). The deduced primer pairs have been tested for their specify by expression analyses. In all cases, fragments of the expected sizes were obtained. Gene-specificity of the primers was further confirmed by the use of nested primers or additional primer combinations. Regarding different isoforms (*AOX* genes) and domain-derived sequence similarities (MADS-box genes), further analysis was performed by sequence-analyses [direct sequencing of PCR fragments obtained from cDNA (MADS-box genes)] or at least by internal PCR-tests with nested primers to ensure a clear differentiation between similar sequences. Flower-specific expression during different developmental stages was analyzed in wild-type carrot plants by RT-PCR or by *in situ* hybridization of mRNA (MADS-box genes, Linke et al., 2003; *AOX* genes, Campos et al., 2009).

**Table 1:** **Primer sequences and amplification conditions for *DcMADS3*, *DcMADS5*, *DcAOX2a*, *DcAOX2b*, and *DcCHS2***.

**Target**	**NCBI number**	**Marker type**	**Forward primer**	**Reverse primer**	**Annealing temperature**	Fragments^*^ detected in the	
						***yel*—mutant**	***cola*—mutant**
*DcMADS3*	AJ271149	EST	GTGTGATGCTAAGGTTTCG	GATCCTGCTCCGCCATG	54	–	1500 bp
*DcMADS5*	AJ271151	CAPS^**^	GGGCACAAAGGAGCTTGAGG	AGAGCATCCACCCTGGAATG	50	605 bp	770 bp
*DcAOX2a*	EU286575	EST	TGCTGCATCTGAGGTCTCTCC	CCAATCAATTCTACACAACAACC	55	1900 bp	–
DcAOX2*b*	EU286576	EST	TGCATGCGTCCTTCCTTATTTTTC	AGCTTTGGTGACAGTATGTATAGG	55	–	1400 bp
*DcCHS2*	D16255	EST	CTCAAGGAGAAGTTTAGGCGGATG	ATGAGGCCATGTACTCGCAGAAA	56	850 bp	900 bp

* *Only polymorphic fragments were summarized. Fragments non-polymorphic between yel- and cola-parent were not mentioned*.

** *The polymorphism was obtained after cleavage of the genomic DNA with Alu I to generate a CAPS (Cleaved Amplified Polymorphic Sequence) marker*.

The different primer pairs were tested for DNA polymorphisms in the *cola*- and the *yel*-parent and in selected progeny plants. PCR amplification products were separated on 1% agarose gels and visualized by ethidium bromide staining. Only primer pairs, providing clear polymorphisms, were used in the segregating F_2_ population DM19. Primers and amplification conditions were summarized in Table 1. Primers for amplification of *DcMADS3* were deduced from NCBI accession number AJ271149. The forward primer binds at the nucleotide positions 114–132 within the MADS-domain. The reverse primer was deduced from the nucleotide positions 653 to 637 that encompassed the last ten amino acids of the coding sequence. For amplification of *DcMADS5*, the forward primer was deduced from the K-box region (LGTK-tag; positions 357–376). The reverse primer binds to the nucleotides 739–720 and was deduced from the PGWML-motif consisting of the five penultimate amino acids preceding the terminus of the predicted protein that reveal similarities within the SEPALLATA1-group of MADS-box genes. For both of the MADS-box genes, unique fragments were obtained that have been classified by PCR-sequencing previously (Linke et al., 2003). The forward primer for amplification of *DcAOX2a* was deduced from the nucleotide positions 539 to 559 of the mRNA-derived cDNA of the mitochondrial alternative oxidase 2a (accession number EU286575.2) in the ferritin-like-diiron-binding region. The reverse primer encompassed positions 1275–1252 and derived from the 3′-UTR region. Two alleles of *DcAOX2a* were known, the L-allele, consisting of 5263 bp (accession number GQ248714) and the S-allele consisting of 4977 bp (accession number GQ248713). Using the described primer set, an L-allele specific fragment of 1930 bp and a S-allele specific fragment of 1654 bp where intron 3 varied in size between 1226 and 941 bp has been characterized (Cardoso et al., 2009). The same gene-specific fragments were also used for analyses of the present mapping population to include these pre-characterized polymorphisms into our yet more extended linkage map. As was shown by Cardoso et al. (2009), the complete gene sequence of *DcAOX2b*, including exons and introns, consisted of 1958 bp. The size of the introns have been assigned to 822 (intron 1), 91 (intron 2), and 85 (intron 3). The length of the deduced mRNA sequence was stated as 1267 bp. For the amplification of *DcAOX2b* we have used primers published by Campos et al. (2009), that covers the nucleotide positions 1–23 (forward primer) of the carrot mitochondrial alternative oxidase 2b (accession number EU286576). The reverse primer was deduced from positions 770 to 747. Forward and reverse primers designed for the amplification of *DcCHS2* were generated from an exon-intron-exon bridge, and were deduced from nucleotide positions 2583 to 2606 and 3484 to 3461 of the gDNA sequence D16255.1, respectively.

Non-polymorphic amplification products were digested with the restriction endonucleases *Alu*I, *Hae*III, *Rsa*I, *Taq*I, *Bsm*I, and *Hinf*I to identify recognition site polymorphisms and to develop CAPS markers by this way.

### Data scoring, linkage analysis, and map construction

The phenotypic and molecular data were formated as required for JoinMap version 4.0 (Van Ooijen, 2006). AFLP-, RAPD-, and dpRAPD-markers were scored in a dominant and SSR-markers in a co-dominant manner. The *p*-value was calculated after chi-square-test for all markers. Linked loci were grouped using a LOD thresholds from 2.0 to 10.0 in steps of 0.2 and recombination frequency lower than 0.4. The jump threshold was set to 5.0 and the third mapping round was carried out. The recombination frequencies were converted to mapping distances (in cM) using the Kosambi function.

## Results

### Phenotype segregation

A homozygous recessive *cola*-mutant plant was used to pollinate a homozygous recessive *yel*-mutant plant to generate the mapping population. All F_1_ plants expressed the wild-type phenotype (green leaves and normal leaf shape). A total of 161 plants of the F_2_ progeny DM19 segregated into four distinct phenotype classes (Figure 1A). The class I plants represent the wild-type of the cultivated carrot. The phenotype class II is highly similar to the wild-type, but the chlorophyll biosynthesis is dramatically delayed resulting in yellowish leaves (*yel*—mutant). Compressed aboveground plant tissues resulting in a semi-dwarf phenotype are the characteristics of the class III plants (*cola*—mutant). Plants of the phenotype class IV showed the characters of both mutants, semi-dwarf plants with yellowish leaves. The visual characters of the roots (size, shape, and color) were not influenced for the different phenotype classes. There was no significant deviation from Mendelian inheritance observed (ratio 9:3:3:1) for two independent loci (Table 2). After vernalization 121 F_2_ plants (75%) flowered within a 100 day period. All plants of the phenotype classes I and II (*n* = 98) developed epigynous flowers, whereas all *cola*-mutant associated plants (phenotype class III and IV; *n* = 23) expressed hypogynous flowers (Table 2; Figures 1B,C).

**Table 2:** **Phenotypical segregation of flower traits in the F_2_ progeny DM19**.

**Phenotypic class**	**No. of plants^a^**	**Observed phenotype**	**Putative genotype**	**Bolting plants^b^**	**Flower development epigynous/hypogynous**
I	90	Green / normal leaves	*YEL*. / *COLA*.	77 (86%)	77/0
II	32	Yellow / normal leaves	*yel yel* / *COLA*.	21 (66%)	21/0
III	31	Green / cola leaves	*YEL*. / *cola cola*	19 (61%)	0/17(2)^c^
IV	8	Yellow / cola leaves	*yel yel* / *cola cola*	6 (75%)	0/6

a *Fit for Mendelian digenic inheritance χ^2^ - (9:3:3:1, phenotype classes) = 0.56; p = 0.91*.

b *Fit for Mendelian monogenic inheritance χ^2^ - (3:1, Bolting: non bolting) = 0.68; p = 0.40; Ratio of bolting plants in the cola classes (III+IV) was significantly lower (64%, p = 0.001) than in the WT (COLA) classes (I+II) with 80%*.

This suggested a potential association to the cola mutant, but a verification by testing of a larger progeny is required;

c *Two plants expressed partially both types of flowers*.

Reduced pollen viability was observed in *cola*-mutant plants in comparison to the wild-type plants (49.9 and 68.7%, respectively). The proportion of small and undeveloped pollen grains was much higher and also the viable pollen grains were significantly smaller than those of the wild type flowers (Table 3; Figure 1D).

**Table 3:** **Microspore characteristics of the *cola*-mutant in comparison to the wild-type**.

**Flower type**	***n***	**Pollen viability^a^ (%) Mean ± *SD***	**S+U Pollen^b^ (%) Mean ± *SD***	**Size of viable microspores^c^**	
				**Length (μm)**	**Wide (μm)**
				**Mean ± *SD***	**Mean ± *SD***
Wild-type (epigynous)	10	68.67 ± 6.89 a	1.36 ± 2.05 a	32.4 ± 5.1 a	16.2 ± 3.1 a
*cola*-mutant (hypogynous)	10	49.88 ± 14.75 b	9.16 ± 5.67 b	28.3 ± 4.2 b	15.5 ± 3.2 a

*n, tested plants, for each plant 400 pollen were evaluated*.

a *Uniformly green-yellow stained pollen*.

b *Number of small (S) and underdeveloped (U) pollen*.

c *Twenty fertile microspores of each were measured. Different letters in a column indicate significant differences (p < 0.05; t-test)*.

### Linkage analysis

**Figure 2:**
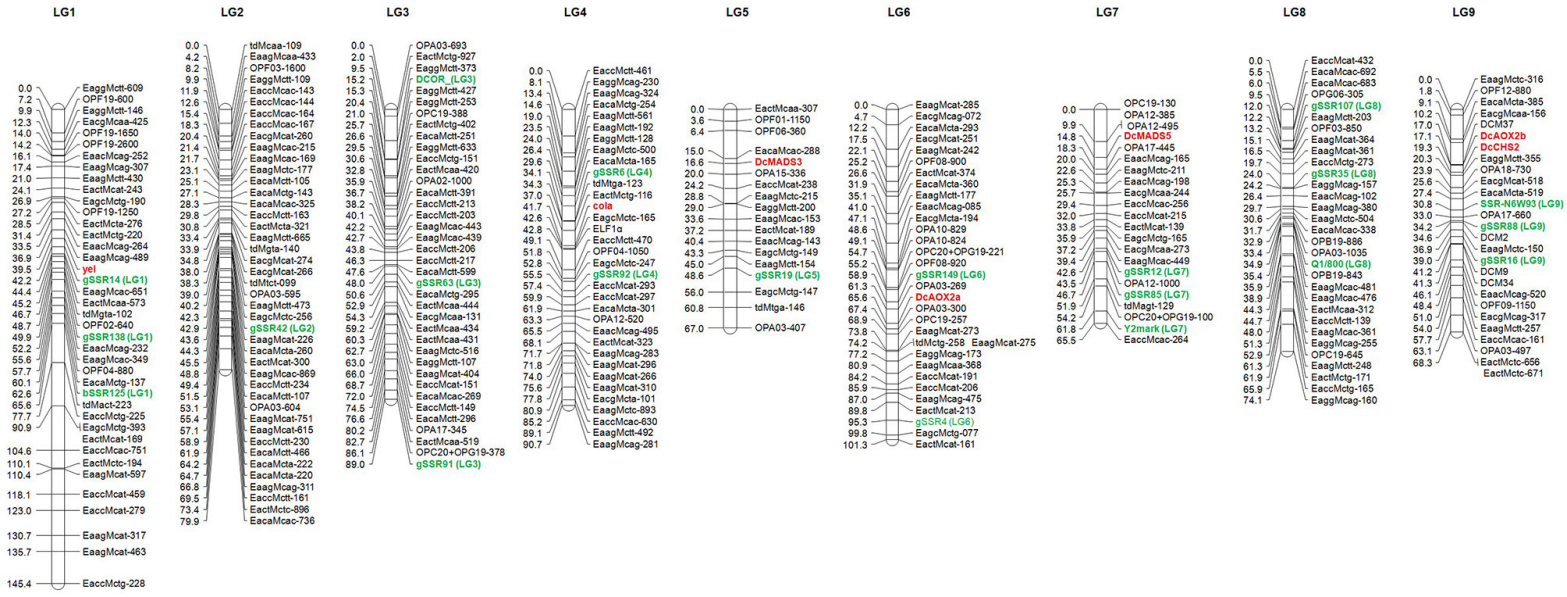
**Genetic map of carrot (*Daucus carota* L.) based on the F_2_ population DM19**. The anchor markers to the carrot reference map (Cavagnaro et al., 2011) are highlighted in green color and designated by the corresponding linkage group number in parenthesis. The newly mapped genes governing flower architecture and pollen development are marked in red. Distances are given on the left of the linkage groups.

After a pre-screening experiment using the parental plants, 21 RAPD primers, 2 dpRAPD, 28 AFLP, and 7 td primer combinations were chosen for the analysis of the segregating population. In total, 319 markers were polymorphic between the parents of DM19 and were used for map construction (Table 4). Nine major groups encompassing 285 markers were generated. The resulting carrot linkage map (Figure 2) had a total length of 781 cM. The number of markers per LG ranged from 18 to 45. The largest minor group, not connected with one of the nine major groups, contained five markers. LG-1 was the largest LG with 145 cM, while LG-7 was the shortest with 66 cM. The average distance between two neighboring markers was 2.7 cM.

**Table 4:** **Summary of mapped markers for the nine linkage groups of the carrot map**.

**Marker**	**AFLP**	**RAPD/dpRAPD**	**td**	**SSR**	**EST/SCAR**	**Mapped traits**	**Total**
Polymorphic mapped	216	60	10	22	9	2	319
	198	45	9	22	9	2	285
LG-1	28	6	2	3	0	*YEL*	40
LG-2	36	3	3	1	0	0	43
LG-3	30	5	0	2	1	0	38
LG-4	27	2	1	2	1	*COLA*	34
LG-5	11	4	1	1	1	0	18
LG-6	20	8	1	2	1	0	32
LG-7	11	6	1	2	2	0	22
LG-8	22	6	0	2	1	0	31
LG-9	13	5	0	7	2	0	27

### Assignment of linkage groups to the reference map

The use of SSR and SCAR markers enabled the LGs of our carrot map to be assigned to the LGs of two reference maps. The original LG names were proposed by Iovene et al. (2011) and we assigned four markers (DcOR, Y2mark, Q1/800, and SSR-N6W93) anchored in their physical carrot map. The LG names were adopted by Cavagnaro et al. (2011) and we were able to map 15 SSRs from LGs 1-9. Two additional SSR markers (gSSR91 and gSSR138) were allocated by Alessandro et al. (2013). The distribution of these 21 markers was generally consistent with the reference maps. Mapping of two or more markers in each LG allowed drawing of seven LGs in the orientation corresponding to that applied by the authors of the reference maps with short/long chromosome arms in the North/South orientation. The orientation of LG-2 and LG-5 remained ambiguous, as each of them was anchored only by one reference marker. Marker gSSR138 was found to be located in our map between gSSR14 and bSSR125 and not distal from gSSR14 as shown by Alessandro et al. (2013).

### Mapping genes responsible for flowering traits

The *COLA*-locus was mapped successfully on LG-4 and the *YEL*-locus on LG-1 of the carrot map. To analyze, whether the flower-specific *cola*-mutant reveal any relationship to one of the flower-specific genes, known to play a prominent role for reproductive organ differentiation and pollen development (Gene-Specific Markers and Table 1), our aim was their preliminary assignment to appropriate LGs. Therefore, we have searched for polymorphisms between the parents of our mapping population. Although phenotypic segregation and our genetic data (compare to PHENOTYPE SEGREGATION) did not favor any association, we have included the data obtained. We have identified sequence polymorphisms for *DcMADS3* and *DcMADS5* as well as *DcAOX2a*, *DcAOX2b*, and *DcCHS2* (Table 1) and mapped them to search for co-localization with the *COLA*-gene.

All of the analyzed gene-fragments revealed a different distribution than the *COLA*-locus, which indicated that there were no associations to the flower-specific defect that has been described here. *DcMADS3* belonging to the B-class of homeotic genes was assigned to LG-5 and *DcMADS5*, a predicted member of the SEPALLATA-group of MADS-box genes, was mapped after cleavage of the amplification product with *Alu*I to LG-7. For *DcAOX2a* a polymorphism was found concerning presence/absence of the fragment specific for the L-allele which was used successfully to assign it to LG-6. In our mapping analyses the polymorphism associated with *DcAOX2b* was assigned to LG-9 indicating a different localization than the *DcAOX2a* sequence.

For the *CHS* gene, the lenght of the obtained fragment polymorphisms corresponded with the predicted size deduced from the genomic sequence of *CHS2* in the *cola*-parent (900 bp), whereas the fragment-length was slightly shortenend in the *yel-mutant* (850 bp).

Despite of this tentative assignment, future work demands a more detailed analysis. Fine-mapping is still required to assign precisely the allelic state and yet undetected potential differences of intron-sizes, like InDels of smaller sizes that could not have been matched in this initial map. For example, a tool for subfamily grouping of the large *AOX* family has been developed, where classification (or re-classification) of *AOX*-subfamily members by specific sequence-features was facilitated (Costa et al., 2014). In summary, all of the five proven genes did not co-segegate with the *cola*-associated flower defects we mentioned in this publication.

## Discussion

To date, a complete carrot genome sequence was not yet available, thus well-saturated genetic linkage maps are of essential relevance for successful carrot breeding and breeding research. Cavagnaro et al. (2011) have developed a linkage map containing 55 SSR markers, offering an important tool for further integration of molecular markers and gene-specific sequences of carrot. Currently, mapping of genes governing traits of economical relevance (e.g., flavors, volatiles and bioactive compounds) in individual well-characterized populations segregating for gene-specific markers is under investigation (Nothnagel, pers. commun.). However, SSR markers offer a useful tool to integrate the results into a consensus map. The total length of the carrot map presented in this work was 781 cM, compared to 669 cM of the map shown by Alessandro et al. (2013), Just et al. (2007), and Cavagnaro et al. (2011) earlier published separated maternal and paternal maps with a total length between 1050 and 1273 cM. The marker density of our map was nearly as high as that presented by Alessandro et al. (2013) and the largest gap was 14 cM. Using SSRs as anchor markers, the LGs of our carrot map matched exactly to the LGs of the reference maps. For seven of the nine LGs at least two anchor markers were allocated enabling us to determine the orientation of the LGs in comparison to the reference maps. Thus, we showed that 21 markers included in the reference maps were suitable for linkage analysis performed on the DM19 population. The carrot populations used for map generation by Cavagnaro et al. (2011), Alessandro et al. (2013) and our group were unrelated and had different pedigrees. Therefore the defined marker set is of fundamental importance for independent mapping projects, as it facilitates the creation of a consensus map.

The *cola*-mutation revealed a compact leaf-habit and a semi-dwarf phenotype that is associated to a reduced cell elongation. Moreover, flowering traits were also affected including an impaired transition to flowering, and abnormal flower architecture with partial male sterility (Nothnagel et al., 2005, this study). The phenotype was inherited in a monogenic recessive fashion causing hypogynous flowers in homozygous state which could be clearly distinguished from epigynous flowers of the wild-type. Additionally, the distorted anther development resulted in lower pollen viability probably due to an impaired development of the sporophytic tissue that surrounded the pollen chambers. The *COLA*-locus was mapped on LG-4.

Although phenotype classification and genetic segregation did not favor the hypothesis, that the *COLA*-locus is related to one of the obtained polymorphisms derived from flower-specific sequences, we have included several of the obtained polymorphisms into our map regarding future aims to extend analyses on flower- and reproductive genes in the carrot. The initiation of flowering and the patterning of floral primordia into discrete domains that give rise to different types of floral organs have been well investigated in model plants. Flower development depends on a complex gene regulation network (Immink et al., 2010; Liu and Mara, 2010). Most of the participating genes encode for transcription factors involved in the regulation of gene expression in a strictly hierarchical manner. A vast majority of the central regulating genes belong to the MADS-box family. Carrot MADS-box genes *DcMADS3* and *DcMADS5* belong to the B-class and E-class MADS-box genes specifying the identity of stamens and hence, the development of anthers and pollen. *DcMADS3* likely plays a similar role in *Daucus* as shown for *Arabidopsis* and *Antirrhinum*. This was supported by the fact that *DcMADS3* was down-regulated in homeotic flowers of the carpeloid CMS type of carrot where stamens were completely replaced by carpels (Linke et al., 2003). For *DcMADS5*, a significant sequence similarity to SEPALLATA1 group and a continuous expression throughout flower development supported the hypothesis that the *SEP1* gene was required from early stages of floral development onwards to mediate activities especially of the B- and C-organ identity genes (Flanagan and Ma, 1994; Pelaz et al., 2000; Honma and Goto, 2001). In the present work, the two carrot MADS-box sequences *DcMADS3* and *DcMADS5* were assigned to LG-5 and LG-7, respectively.

Campos et al. (2009) have shown differential expression of *DcAOX1* and *DcAOX2* genes at early stages of floral organ formation. As reported by Cardoso et al. (2009), *AOX2a* was successfully analyzed in a preliminary mapping approach of the DM19 population. In the present work, obtained polymorphisms for *AOX2a* and *AOX2b* were assigned to the LGs LG-6 and LG-9, respectively. Taylor and Jorgensen (1992) demonstrated that the development of the male gametophyte essentially required flavonoid biosynthesis. Hence, CHS deficient plants (with so-called white pollen) were self-incompatible. Pollen germination on the stigmata could be restored when a small amount of kaempherol was provided (Mo et al., 1992). In this study, a *CHS* sequence of *D. carota* was assigned to LG-9.

In conclusion, a well-saturated map of carrot was developed. The *cola*-locus, associated to advanced defects of male and female organ differentiation was associated to LG-4 and revelaed no co-segration with several markers for genes involved in flowering and reproduction. Hence, six loci significant for these processes were mapped to five LGs. Considering two other genes, *Vrn1*, responsible for early flowering habit and *Rf1* restoring petaloid CMS, have been assigned by Alessandro et al. (2013) to LG-2 and LG-9, respectively, it can be assumed that eight loci associated with flower architecture and reproduction are dispersed to six out of nine carrot chromosomes. Indeed, fine-mapping of the identified loci will be required, especially regarding the growing number of subgroup members within the selected gene-families that are not yet completely available in the database. Several tools for structural analyses of high diversity of gene families, like the compositions of the *AOX* genes might facilitate a detailed analysis also in non-model plants (Costa et al., 2014).

Finally, the use of anchor markers that exclusively derived from the American gene pool, enables the generation of the first integrated carrot map based on markers of both, the European and American carrot gene pool. This opens advanced options for the establishment of a comprehensive consensus map by co-operative activities between the research groups working on carrot genetics. Furthermore, the addition of gene-derived markers to the present map may provide a good starting point for comparative mapping in other Apiaceae species like *Apium*, *Petrosellinum*, *Carum*, or *Foeniculum* for which *Daucus* can serve as well-characterized representative. This might facilitate the future research on genes associated with traits important for both, food and non-food sector (Barańska et al., 2005; Ulrich et al., 2011) and allows the identification of genes with general interest for Apiaceae species.

## Author contributions

Holger Budahn: SSR analysis and map construction. Rafał Barański: AFLP and SSR analysis. Dariusz Grzebelus: td analysis. Agnieszka Kiełkowska: RAPD and AFLP analysis. Petra Straka: RAPD and AFLP analysis. Kai Metge: RAPD and AFLP analysis. Bettina Linke: Primer design MADS-box genes, PCR-analyses of parental plants of the mapping population, substantial contributions to conception and design of the manuscript. Thomas Nothnagel: Generation of the mapping population, flower morphology, histological analysis, substantial contributions to conception and design of the manuscript.

## Funding

Parts of this work were supported by a bilateral cooperation project of BMELV (Germany) and MNiSW (Poland, project DS3500/KGHN granted to the Univ. of Agric. in Krakow).

### Conflict of interest statement

The authors declare that the research was conducted in the absence of any commercial or financial relationships that could be construed as a potential conflict of interest. Furthermore, we declare that the experiments comply with the current laws of Germany and Poland.
